# Automated Laboratory Reporting of Infectious Diseases in a Climate of Bioterrorism

**DOI:** 10.3201/eid0909.020486

**Published:** 2003-09

**Authors:** Nkuchia M. M’ikanatha, Brian Southwell, Ebbing Lautenbach

**Affiliations:** * Pennsylvania Department of Health, Harrisburg, Pennsylvania, USA; † University of Minnesota, Minneapolis, Minnesota, USA; ‡ University of Pennsylvania School of Medicine, Philadelphia, Pennsylvania, USA

**Keywords:** Computerized data collection, disease notification, epidemiologic methods, medical records, population surveillance, public health, public policy, new media

## Abstract

While newly available electronic transmission methods can increase timeliness and completeness of infectious disease reports, limitations of this technology may unintentionally compromise detection of, and response to, bioterrorism and other outbreaks. We reviewed implementation experiences for five electronic laboratory systems and identified problems with data transmission, sensitivity, specificity, and user interpretation. The results suggest a need for backup transmission methods, validation, standards, preserving human judgment in the process, and provider and end-user involvement. As illustrated, challenges encountered in deployment of existing electronic laboratory reporting systems could guide further refinement and advances in infectious disease surveillance.

The primary purpose of reporting diseases is to trigger an appropriate public health response so that further illness can be prevented and public fears allayed. The threat of emerging infections and bioterrorist attacks has heightened the need to make disease surveillance more sensitive, specific, and timely (*1*,*2*). Recent advances in provider and laboratory information management have facilitated one step towards the modernization of surveillance: the development of automated reporting systems (*3*,*4*). With recent funding for activities to defend the public’s health against terrorism and naturally occurring diseases, development of automated reporting system has been accelerated (*5*).

However, technologically innovative reporting systems need to be consistent with the purpose of disease reporting. Wholesale adoption of automated electronic reporting systems in their current form might instead represent a quick response to the pressures of the moment rather than a fully considered decision that acknowledges some of the documented problems with the new technology. We review here current limitations of systems that provide automated notification of reportable conditions identified in clinical laboratories. A more thorough understanding of the pitfalls of such existing systems can provide insights to improve the development and implementation of new media in infectious disease surveillance.

With the computerization of patient and clinical laboratory data, automated notification of reportable events to health departments is often assumed to be more effective than conventional paper-based reporting (*6*). In recent years, the Centers for Disease Control and Prevention (CDC) has been funding several states to develop electronic laboratory reporting (*7*). With electronic reporting, laboratory findings (e.g., *Escherichia coli* O157:H7 isolates) are captured from clinical laboratory data and transmitted directly to the state. In turn, the state routes messages to local health units, as illustrated in the Figure. The National Electronic Disease Surveillance System (NEDSS) and bioterrorism preparedness initiatives are expected to further enhance disease surveillance by supporting integration of electronic data from various sources (*4*,*8*). Evidence from deployed systems shows promise in the ability of electronic laboratory reporting to deliver more timely and complete notifications than paper-based methods (*9*–*12*).

At the same time, experiences in Pennsylvania, New York, Hawaii, California, and other states indicate that implementation of automated reporting also poses unanticipated challenges. Five problem areas have been identified: sensitivity, specificity, completeness, coding standards, and end-user acceptance.

## Sensitivity

To achieve the objective of triggering local public health response, automated electronic systems should consistently report cases that would have been reported by conventional methods. Contrary to expectations, automated reports seldom replicate the traditional paper-based system. Errors in data transmission reduce sensitivity in automated electronic reporting systems. An evaluation of electronic laboratory reporting in Hawaii documented that automated reports were not received for almost 30% of the days on which the paper-based method generated a report, suggesting that automated reporting alone was potentially suboptimal. Lapses in electronic reporting were due to various causes including ongoing adjustments to the data extraction program (*11*). In California, lapses in a semiautomated electronic laboratory reporting were traced to a failure in forwarding reports from the county of diagnosis to the county of residence (*12*). In Pennsylvania, lapses in automated notification have resulted from the occasional failure of data extraction at the clinical laboratory computer, difficulties deciphering reportable diseases from the test results, which used local terminology rather than Logical Observation Identifier Names and Codes (LOINC) codes (available from: URL: http://www.regenstrief.org/loinc), and problems in the transmission of data files to and access by local health jurisdictions. To prevent interruption of reports while the automated system was being refined, Pennsylvania opted to continue conventional paper-based reports for 8 months after initiating electronic reporting.

### Specificity

Typically, automated reporting increases not only reportable events data but also the number of extraneous reports (e.g., nonreportable conditions, unnecessary negative reports, or duplicate reports). In addition, false-positive results are increased by automated abstraction of culture results entered in free-text. For example, in an evaluation of an electronic laboratory reporting system in Allegheny County, Pennsylvania, negative results of *Salmonella* isolates were automatically transmitted as positive *Salmonella* results because the software recognized the organism name (*9*). Often, automated reporting transmits preliminary test results followed by results of confirmatory tests for the same condition. This method is desirable because some duplicates may actually provide useful preliminary test results that might trigger timely responses (*9*,*10*). However, multiple test results increase time for data processing. In addition, low specificity attributable to extraneous records of nonreportable culture results is also problematic. While over time automated programs can be expected to improve, initially erroneous or missing data will continue to arise and require manual checking and recoding.

Programming solutions might offer relief in eliminating extraneous records. But in a climate of bioterrorism, a complete replacement of human judgment is probably unacceptable for many. Therefore, in planning new systems, accounting for the time and effort of an experienced epidemiologist to review electronic laboratory data before routing them to investigators will be essential.

### Completeness of Case Records

To be useful, case-reports received through conventional or automated methods must contain data in key fields identifying patient and physician (e.g., name, address, and telephone number) and specimen (e.g., collection date, type, test, and result). Lack of sufficient identifying information for follow-up investigations is a serious limitation in many currently operating automated systems.

In addition, experiences in New York and Pennsylvania indicate that the lack of a patient’s address is a barrier to routing electronic laboratory data to local health departments. Locating a patient’s residence is also useful for recognizing clusters of diseases attributable to natural causes or intentional acts of terrorism. Automated means were intended to improve completeness of case records fields by duplicating required fields, but this has not always been the case (*13*). Whether the laboratories fail to report missing data or whether data elements are not provided in the initial forms submitted with specimens is unclear. Widespread dissemination of standardized disease reporting forms specifying information required by health departments to both clinical laboratories and providers could reduce this problem. Such information could also be made readily available through the Internet. An example is of what laboratories and providers are required to include in Minnesota is available (URL: http://www.health.state.mn.us/divs/dpc/ades/surveillance/card.pdf).

### Data Standards

To facilitate use of state-of-the-art electronic surveillance tools, as envisioned in the NEDSS initiative, adoption of Systemized Nomenclature of Human and Veterinary Medicine (SNOMED) (available from: URL: http://www.snomed.org/), LOINC, and Health Level 7 standards (a national standard for sharing clinical data, available from: URL: http://www.hl7.org/) by clinical laboratories is essential. However, in practice clinical laboratories often use locally developed coding schemes or a combination of codes and free text. Data often arrive in multiple file formats or even with multiple formats within one mapping standard (Figure). In practice, file messages from multiple laboratories are mapped into a standardized database with desired variables including patient, physician contact information, specimen identifiers, test name, and results.

**Figure:**
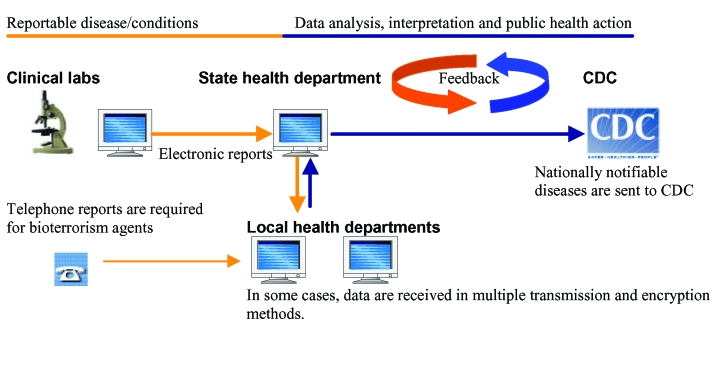
Steps in automated reporting of infectious disease data. The process begins with abstraction of reportable conditions using a software program. Data are stored in a file for future transmission or sent directly to the health department in the case of automated reporting systems. Typically, there are multiple clinical laboratories, and reports are transmitted in a variety of methods including file transfer protocol and dial-up modem at arranged intervals. State health departments review data and forward them to local health departments, where investigations are done and reportable conditions are determined. Local health departments forward data back to the state, where further analysis and interpretation are accomplished. The state uploads nationally notifiable diseases data to a secure data network at the Centers for Disease Control and Prevention. That agency sends data quality feedback to the state immediately. The level of feedback among states, laboratories, and local health departments is unknown but suspected to vary widely.

To increase use of uniform data coding and Health Level 7 as the standard for automated electronic reporting, further studies are needed to understand barriers encountered by clinical laboratories and ways to overcome them. Cost or lack of information technology resources might be factors contributing to slow adoption of standard coding in small-size clinical laboratories. In addition*,* variations in reporting requirements across states may be an extra cost to laboratories that serve multiple health jurisdictions. Public health officials could help promote use of coding standards by demonstrating their benefits to laboratories and providers. For example, use of standards such as LOINC facilitates integration of microbiologic culture data, minimizes chances for data errors in translating free text or handwritten test results, and makes it easier for laboratories to monitor antimicrobial resistance patterns. This could be reinforced by introducing regular data quality feedback to all the stakeholders, as illustrated in the Figure.

### User Acceptance

This entire process for detecting diseases relies on acceptance and appropriate intervention by those working on the front-line of the public health system. As shown on the Figure, public health surveillance largely depends on investigation at the local level, where a determination is made that reported events meet case definitions for reportable and notifiable conditions. Local health departments report data to the state level, where nationally notifiable diseases (available from: URL: http://www.cdc.gov/epo/dphsi/phs/infdis.htm) are transmitted to CDC. That agency in turn reports internationally quarantinable diseases to the World Health Organization (available from: URL: http://www.who.int/emc/IHR/int_regs.html). The process begins with receiving, managing, and using surveillance data. Automated reports in the form of electronic-mail attachments could be cumbersome for some local health departments with limited information technology support. Also, encryption of data for confidentiality reasons increases complexity of the data retrieval process. Acceptance of automated electronic reporting systems is likely when assistance on data analysis and management is given to disease investigators.

During the 2001 bioterrorism outbreak investigation, labor-intensive methods (i.e., faxes and emails) were used for surveillance of cases with clinical syndromes compatible with anthrax among patients in selected counties in New Jersey, Pennsylvania, and Delaware (*14*). Because of personnel time demands, automated electronic systems are attractive in surveillance of syndromes suggestive of bioterrorism agents. While automated electronic surveillance systems using patient encounter records for syndromic surveillance might offer relatively low costs of adoption for physicians (*15*), other persons in the system may become unduly burdened. For example, when automated reports of syndromes are forwarded to local public health officials, who should interpret and act upon the results remains unclear. The key to the success of such innovative systems outside investigational settings will be their ability to offer meaningful results at an acceptable marginal cost to both reporters and local health departments. Integration of syndromic surveillance into local public health surveillance is less understood and needs attention.

## Discussion

Responding to and anticipating the difficulties encountered by existing automated reporting systems could be used to improve current systems and guide development of future infectious disease surveillance. Addressing limitations of automated reporting systems by continuing conventional notification methods during the adjustment period, promoting use of coding standards, validating data, and involving end-users is essential.

As illustrated in this study, lapses in data transmission occur during initial deployment of automated reporting systems. The potential risks attributable to lapses or errors in automated electronic reports are great, as are costs associated with misdiagnoses and treatment of healthy persons (*16*). Experiences in Hawaii and Pennsylvania indicate the need for continuing with existing reporting mechanisms during the first year while new systems are being refined.

Our study calls for evaluations to validate new automated systems before they are integrated into public health surveillance. While health departments and CDC have typically collaborated in such efforts, involvement of providers and laboratorians is likely to yield additional insights. Participation of public health officials is indicated in evaluations of automated methods that are being developed in research settings to capture nonreportable syndromes for bioterrorism detection.

Partnerships among state health departments, clinical laboratories, providers, CDC, and other diagnostics systems are needed to promote widespread use of uniform coding standards (LOINC and SNOMED) and Health Level 7 for messaging. As demonstrated in New York State, involving all users early in the planning stages enhances the success of automated electronic reporting system (*13*). CDC could facilitate laboratory participation in use of standards by assisting health departments in identifying benefits such as use of LOINC-coded data for antimicrobial resistance monitoring.

Current federal funding for emergency preparedness surveillance and epidemiology capacity (*17*) is expected to stimulate widespread use of automated systems in infectious reporting. However, automated systems are a complement rather than a substitute for human involvement in interpreting laboratory findings and screening for errors. Furthermore, the requirement that providers and laboratories report immediately by telephone when they detect organisms indicating an outbreak or an unusual occurrence of potential public health importance (*18*) is expected to continue even when automated reporting systems are implemented. Complete replacement of human judgment in reporting conditions suggestive of CDC category A bioterrorism agents (available from: URL: http://www.bt.cdc.gov/Agent/Agentlist.asp) or other conditions that require immediate investigation is unrealistic.

Despite the limitations we have described, automated electronic systems hold promise for modernizing infectious disease surveillance by making reporting more timely and complete. Modern technology can translate into better public health preparedness by enhancing and complementing existing reporting systems.
